# Insights From Dynamic Neuro-Immune Imaging on Murine Immune Responses to CNS Damage

**DOI:** 10.3389/fnins.2019.00737

**Published:** 2019-07-17

**Authors:** R. Dixon Dorand, Bryan L. Benson, Lauren F. Huang, Agne Petrosiute, Alex Y. Huang

**Affiliations:** ^1^Department of Medicine, Vanderbilt University Medical Center, Nashville, TN, United States; ^2^Department of Pathology, Case Western Reserve University School of Medicine, Cleveland, OH, United States; ^3^Department of Pediatrics, Case Western Reserve University School of Medicine, Cleveland, OH, United States; ^4^Angie Fowler Adolescent & Young Adult (AYA) Cancer Institute/University Hospitals (UH) Rainbow Babies & Children’s Hospital, Cleveland, OH, United States

**Keywords:** two-photon microscopy, microglia, CNS, immune response, innate and adaptive immunity, tissue microenvironment, blood-brain-barrier, vascular crawling

## Abstract

Evolving technologies and increasing understanding of human physiology over the past century have afforded our ability to intervene on human diseases using implantable bio-materials. These bio-electronic devices present a unique challenge through the creation of an interface between the native tissue and implantable bio-materials: the generation of host immune response surrounding such devices. While recent developments in cancer immunology seek to stimulate the immune system against cancer, successful long-term application of implantable bio-material devices need to durably minimize reactive immune processes at involved anatomical sites. Peripheral immune system response has been studied extensively for implanted bio-materials at various body sites. Examples include tooth composites (Gitalis et al., 2019), inguinal hernia repair (Heymann et al., 2019), and cardiac stents and pacemaker leads (Slee et al., 2016). Studies have also been extended to less well-studied immune reactivity in response to CNS neural-electronic implant devices. Recent technological advances in 2-Photon Laser Scanning Microscopy (2P-LSM) have allowed novel insights into *in vivo* immune response in a variety of tissue microenvironments. While imaging of peripheral tissues has provided an abundance of data with regards to immune cell dynamics, central nervous system (CNS) imaging is comparatively complicated by tissue accessibility and manipulation. Despite these challenges, the results of dynamic intravital neuro-immune imaging thus far have provided foundational insights into basic CNS biology. Utilizing a combination of intravital and *ex vivo* 2P-LSM, we have observed novel pathways allowing immune cells, stromal cells, cancer cells and proteins to communicate between the CNS parenchyma and peripheral vasculature. Similar to what has been reported in the intestinal tract, we have visualized myeloid cells extend dendritic processes across the blood brain barrier (BBB) into pial blood vessels. Furthermore, transient vessel leaks seen during systemic inflammation provide opportunities for cellular protein to be exchanged between the periphery and CNS. These insights provide new, visual information regarding immune surveillance and antigen presentation within the CNS. Furthermore, when combining intravital 2P-LSM and microfluidic devices complexed with mathematical modeling, we are gaining new insights into the intravascular behavior of circulating immune cells. This new knowledge into the basic mechanisms by which cells migrate to and interact with the CNS provide important considerations for the design of neuro-electronic biomaterials that have the potential to connect the peripheral-neural microenvironments into a unique, artificial interface.

## Introduction

The use of implantation devices dates back to ancient Egypt with archeological discovery of a toe prosthesis (Nerlich et al., 2000; Brier et al., 2015). More recently, technological advances have allowed clinical application of implantable electronic devices such as pacemakers and deep brain stimulators, once thought to belong to the realm of science fiction, to become routine in clinical practice. These devices have advantages due to their macroscopic scale and relatively large output voltages, which allow them to overpower the potential negative functional effects of tissue fibrosis and host immune reactions in response to the presence of foreign substances.

Meanwhile, challenges remain in the field of implantable neural devices, whether in applying fine sensor arrays for recording neural activity, or in reconstituting neural activities into motor commands to circumvent deficits resulting from spinal cord injury or stroke. Numerous factors contribute to this ongoing challenge, but two are classically identified as being of major importance: tissue stiffness mismatch and breakdown of blood-brain barrier (BBB). Critically, both of these issues interrupt normal neural physiology, implying that simple strategies to prevent tissue fibrosis, such as scavenging of cytokines like TGF-β, will not be sufficient. Such an approach may reduce impedance from tissue fibrosis, but would result in faithfully reporting deranged neural activity.

First and foremost, these devices do not match tissue stiffness of the brain, an organ with the lowest stiffness second only to blood. The importance of tissue stiffness is underscored by the broad success of bony prostheses dating back before recorded history, whereas for blood, the most pliable tissue, even state-of-the art intravascular devices such as extracorporeal membrane oxygenation remain plagued by tissue reaction. This issue is well established in neural technology and extensively reviewed elsewhere (Lotti et al., 2017; Salatino et al., 2017; Campbell and Wu, 2018).

Second, and unique to brain tissue, device implantation invariably disrupts the BBB. This disruption leads to dramatic alterations in the function of all intra-parenchymal cells, whose physiology is predicated on residing within an environment separated from the intravascular milieu. Microglia, astrocytes, neurons, pericytes, and brain endothelial cells are all known to trigger inflammatory programs in response to serum proteins present within the brain parenchyma. In turn, this leads to further inflammation as intra-parenchymal cytokine release promotes further increase in BBB permeability, resulting in accumulation of peripheral blood immune cells such as monocytes, a cell type that is typically excluded from the immune-privileged brain parenchyma. This inflammation occurs via the coordinated synergistic efforts of multiple mechanisms, most of which follow patterns well established in other tissues.

## Failure of Neuro-Electronic Devices

In a quest to restore function to damaged neuronal circuits resulting from trauma, stroke, or neurodegenerative processes, implantable neuro-electronic devices have shown some efficacy. However, the success of these neuro-electronic devices have been limited by inflammatory responses, scarring, and associated neuronal cell death in response to immune cell activation, leading to a progressive neurodegenerative state and ultimate failure of device function (McConnell et al., 2009). In one study, of 78 silicon microelectrode arrays (MEA) implanted in 27 non-human primates, 56% of devices failed within 1 year with a mean recording duration of 387 days. Approximately 15% of devices failed due to the development of a dense, fibrous meningeal encapsulation with extrusion of the device from the cortex (Barrese et al., 2013). Additional studies demonstrated that CCL2- and TNFα-secreting CD68^+^ cells were responsible for MEA device failure that was associated with the destruction of neuronal circuits in close proximity to the implanted bio-materials (Biran et al., 2005). More recently, peripheral blood-derived monocytes bearing surface CD14 antigen has been implicated as a dominant innate immune cell population that could potently mediate neurodegenerative pathologies associated with microelectrode implantation (Ravikumar et al., 2014b), and serve as a potential therapeutic target to alleviate this problem (Bedell et al., 2018; Hermann et al., 2018). Further review of the acute and chronic tissue response to electrode implantation is reviewed elsewhere (Fernandez et al., 2014; Kozai et al., 2015).

## Immune Cells in the CNS

It is critical to decipher which cell types are responsible for generating and potentiating immune responses in the CNS in order to potentially address the issue of immune-mediated device failure. In peripheral tissues, dendritic cells (DCs) and circulating monocytes serve as the major source of antigen presenting cells (APCs), which recognize damage-associated molecular patterns (DAMPs) or pathogen-associate molecular patterns (PAMPs), while presenting processed antigenic peptides in the draining lymph nodes (Itano and Jenkins, 2003). However, in the CNS, APCs are made up of both CNS-resident microglia and blood-derived DCs (Ransohoff and Cardona, 2010). Microglia, characterized as CX_3_CR_1_^+^/Ly6C^lo^/CD45^lo^/Iba-1^+^, serve as resident phagocytes and immune sentinels of the non-inflamed CNS parenchyma, as they utilize their dendritic extensions to survey the entire CNS multiple times per day (Nimmerjahn et al., 2005). Microglia are present in both white and gray matter in varying densities and may possess different activation states based on their anatomic location (Lawson et al., 1990; Mittelbronn et al., 2001). Microglia have been shown to play a functional role in enhancing the activity of neuronal synapses through physical contacts. Disruption of these synapses by inflammatory stimuli could result in dis-synchronization of neural networks (Akiyoshi et al., 2018). Because of these important Microglial functions, understanding microglia physiology is essential as they are major contributors to the success or failure of implantable bio-materials. Microglia begin to show morphologic changes from as far as 130 microns from probe insertion sites in as soon as 6 h post implantation (Kozai et al., 2012b), implying that these immune cells’ reactivity may extend beyond direct physical contact.

## Strategies for Modifying Microglia Function

Since microglia play an integral role in the CNS immune response, both biological interference and mechanical device design play a central role in manipulating the neuro-electronic interface. For example, anti-Parkinson’s disease monoamine oxidase B inhibitors have been shown to dampen the inflammatory response of microglia by regulating voltage-gated sodium channels, reducing the neuro-inflammatory reaction to MPTP (Hossain et al., 2018). Another novel target includes the THIK-1 potassium channel, which was recently shown to play an integral role in microglia ramification, surveillance, and was shown to be necessary for IL-1b release. Injection of tetrapentylammonium reduced microglia surveillance by 60% (Madry et al., 2018). Additionally, coating electrodes with neuronal adhesive molecules can help dampen the acute effects of the innate immune response by 80% (Eles et al., 2017). Lastly, reducing electrode size and enhancing their mechanical compliance with brain tissue also reduces the immune response to implanted bio-materials, as evidenced by diminished microglia and astrocyte activation (Kozai et al., 2012a). Whether these methods of immune suppression will lead to overall improved device longevity and decreased scar formation remains to be seen. Nevertheless, these and other approaches certainly bear further investigation.

## Peripheral-Derived Myeloid Cells

Another important immune cell type to consider is the CX_3_CR_1_^+^/Ly6C^+^/CD45^hi^/CCR2^+^ blood derived macrophages that can be found in the perivascular and parameningeal spaces and can sample cerebrospinal fluid (CSF) in the arachnoid and Virchow-Robin spaces (McMahon et al., 2006; D’Agostino et al., 2012). Peripheral monocytes can be further divided into an inflammatory subset (CX_3_CR_1_loCCR2hiGR1+Ly6Chi) that are short-lived and home to inflamed tissue, or a patrolling subset (CX_3_CR_1_hiCCR2loGR1-Ly6Clo) that are long-lived and are recruited to non-inflamed tissue (Geissmann et al., 2003). Common to microglia and both monocyte subsets is the surface expression of CD14, a glycosylphosphatidyl-inositol-anchored protein notable for its role as a co-adaptor protein for toll-like receptor 2 (TLR-2), toll-like receptor 4 (TLR-4), and damage-associated molecular patterns (DAMPS) including heat shock protein 70 (hsp70) and S100A9 (Haziot et al., 1988; Yang et al., 1999; Asea et al., 2000; Beschorner et al., 2002; He et al., 2016). Using bone marrow chimera approach to distinguish the contribution of brain-resident microglia versus bone marrow-derived infiltrating monocytes, we observed that blood-derived monocyte/macrophages, not CNS-resident microglia, dominated the infiltrating cell population following microelectrode implantation site at 2 and 16 weeks post implantation, and their densities correlated with neuron loss at the microelectrode-tissue interface (Ravikumar et al., 2014b). In particular, functional CD14 on blood-derived monocytes and the presence of endotoxin contamination on intracortical microelectrode devices contribute to the decline in overall device performance (Ravikumar et al., 2014a; Bedell et al., 2018), so much so that specific inhibition of CD14 signaling pathway were shown to improve long-term performance of the implanted microelectrode (Hermann et al., 2018).

## Application of Intravital 2P-LSM to Study the CNS

As host immune response is a dynamic process involving the recruitment, retention, and functional differentiation of highly motile immune cells to damaged CNS tissue sites, traditional static histologic examinations often do not fully capture the evolution of the inflammatory process. To this end, 2P-LSM allows for the visualization of fluorescently tagged structures deep within undisturbed living tissues in a time-resolved, dynamic fashion (Denk et al., 1990). Two photon excitation also enables second harmonic generation to illuminate ordered structures such as collagen and myosin without exogenous fluorescence probes, thus allowing for morphologic observation in collagen-rich structures within tissues such as the lymph node and the brain (Campagnola et al., 2002; Williams et al., 2005; Kawakami and Flugel, 2010). While the field of immunology has taken advantage of two photon technology in the past two decades (Germain et al., 2012), neuroscientists first began using this technology in the late 1990s to monitor calcium flux in organotypic brain slice cultures (Yuste and Denk, 1995). Since the early 2000s, intravital CNS imaging in the brain has flourished and scientists have been able to capture a variety of biological processes including but not exclusively: astrocyte reactivity, dendritic spine turnover, formation of plaques in Alzheimer disease, microglial dynamics, and immune cell trafficking. Using intravital 2-photon laser scanning microscopy (2P-LSM), our group has identified three additional mechanisms of immune surveillance in various physiological and pathological states that may have unique implications for brain tissue reactivity to intracortical implantation devices.

For the remainder of this review, we will focus on these novel insights provided by a combination of disease models and application of intravital 2P-LSM to reveal synergistic mechanisms that maintain BBB disruption and promote ongoing inflammation within the brain parenchyma. First, similar to what has been reported in the intestinal tract, we have visualized inflamed myeloid cells extending dendritic processes across the BBB into pial blood vessels. Second, we have demonstrated that cytokine-induced BBB permeability increase is temporal-spatially heterogenous and includes prominent but transient vessel leaks. Third, we have demonstrated that geometric changes to the pial vasculature in response to inflammation promote the arrest of peripheral blood immune cells, promoting their recruitment into the brain. We propose that these additional mechanisms must be addressed to achieve faithful, fail-safe and durable recording and control by neural implants.

## Intravascular Dendritic Projections by Myeloid Cells Through Intact BBB

Recently, we described a unique mechanism whereby microglia extend their dendritic processes through the basement membrane and endothelium into the surrounding pial vessels, thereby providing a new potential avenue for immune cell interactions (Barkauskas et al., 2013). While extending dendritic processes between distinct anatomic compartments is not unique to the CNS (Niess et al., 2005; Girard et al., 2012), this is the first-time microscopy has captured this type of interaction between the immune privileged CNS and peripheral tissues. Using electron microscopy, we were able to confirm astrocyte end feet on either side of the extension, indicating that the astrocytes are actively participating in this breach of the BBB. At baseline in the gray matter of the cortex, we found a density of 175 CX_3_CR_1_^+^ cell projections per mm^2^ of vessel wall surface compared with only 75 projections in the dorsal column white matter in the spinal cord. Using three distinct pathologic models, we compared the frequency of projections in different inflammatory states. During the induction phase of experimental autoimmune encephalomyelitis (EAE), we found that the number of extensions doubled in the cortex by day 9 of induction and doubled in the spine by day 12, even though clinical symptoms usually only appear by day 12. In the cortex, projections could be seen in vessels of both small and large caliber, while in the spinal cord, they were only seen in association with small caliber vessels. In contrast to this EAE model, studies of traumatic spinal cord injury showed a 40% reduction in projections that slowly recovered to 85% of baseline by day 8 after injury. Projections in spinal cord injury were associated with both large and small caliber vessels. Of interest, in a third model of primary CNS malignancy in the gray matter of the cortex, we observed dendritic extensions into the tumor neo-vasculature with a frequency of less than half of baseline (73/mm^2^ vs. 175/mm^2^). These intriguing observations required further investigations in order to understand the molecular and cellular signaling between microglia, surrounding CNS parenchyma, and endothelial tissues that regulate this behavior under physiological and different disease states.

Differential contribution of resident microglia and blood-derived monocytes/macrophages to neuron loss during CNS trauma was also demonstrated by direct intravital observation by 2P-LSM. Using a traumatic spinal crush injury model, we investigated the role of inflammatory monocytes in the process of post-traumatic axonal dieback (Evans et al., 2014). Immediately after crush injury, CX_3_CR_1_+ cells extravasated from blood vessels into the lesion and increased their abundance by six-fold in 5 days. Parenchymal microglia, on the other hand, did not accumulate at the injury site. While imaging animals on days 2, 5, and 8 post-injury, we observed axon-macrophage contacts that were associated with morphologic changes of the axon, ultimately resulting in either thinning of the axon or destruction within an hour.

## Transient Cns Vessel Leak and DC Accumulation

Peripheral monocytes can also differentiate into DCs, characterized as CD11c+, and have the potential to stimulate CD8+ T-cells (Geissmann et al., 2003). Using an EAE model, we utilized intravital 2P-LSM to elucidate the initiating events in EAE and found that in fact multiple cell types are required to induce durable immune responses. First, we found transient focal blood vessel leaks from post-capillary venule junctions that occur within hours to days immediately following EAE induction before any clinically evidence symptoms can be appreciated. These leaks lasted as long as 30 min and where rapidly cleared by CSF flow near the pial surfaces but persisted when vessel contents leaked into the parenchyma. We visualized CX_3_CR_1_ microglia phagocytose blood vessel contents, which lead to their activation in the first 3 days following induction of EAE. The phagocytic CX_3_CR_1_+ microglia were then associated with an increase in CD11c+ DCs by day 6 post induction, which was then followed by infiltration and accumulation of antigen-specific T cells by day 9. Both antigen-specific T-cells and DCs congregated around areas of previous vessel leak, forming immune clusters within the CNS parenchyma. Following these observations of immune clusters, clinical EAE symptoms then started around day 10–12. Of interest, treatment with hydroxyzine ultimately decreased microglia activation and phagocytic capacity by day 3 and resulted in decreased accumulation of peripheral immune cells by day 6 (Barkauskas et al., 2015).

**FIGURE 1:**
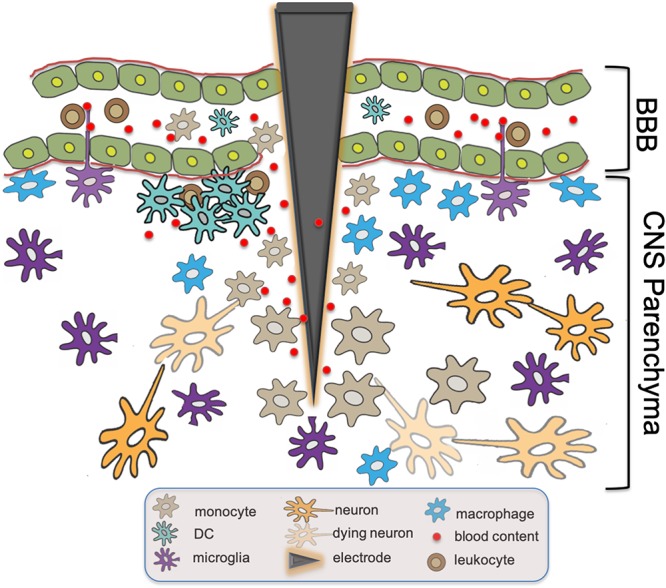
Innate immune responses at intracortical electrode implantation sites. BBB Breach resulting from trauma associated with the insertion of intracortical electrodes causes leakage of blood content into CNS parenchyma. Subsequent tissue inflammatory response includes the recruitment of monocytes expressing CD14,the accumulation of DCs and macrophages, as well as the activation of microglia with intravascular dendritic protrusions that potentially participate in the further recruitment of circulating immune effector cells. The presence of DAMPs activates these innate immune cells via molecular signaling cascades including CD14, resulting in the eventual neuron loss and failure of the electronic device over time.

BBB Permeability has been implicated in the progression of multiple pathologic processes including Alzheimer’s disease (AD), psychosis, and systemic lupus erythematosus (Bowman et al., 2007; Hirohata et al., 2018; Pollak et al., 2018). Indeed, an inverse correlation has also been demonstrated between CNS electronic implant performance and the severity and frequency of BBB breach (Saxena et al., 2013; Fernandez et al., 2014). Histamine, bradykinin, fibrin, and clotting pathways have all been implicated in modulating BBB permeability (Ryu and McLarnon, 2009; Passos et al., 2013; Chen et al., 2017). In fact, the accumulation of CNS infiltrating DCs was further demonstrated recently in a murine model of Alzheimer’s disease, a process that was directly related to plasma Factor XII. Reduction in Factor XII correlated with reduced inflammation, neuronal damage, and improved cognitive function in the early stages of AD (Chen et al., 2017). Utilizing intravital 2P-LSM in these disease models would be helpful in establishing whether similar vessel leaks as observed in our EAE studies (Barkauskas et al., 2015) could also serve as a primary mechanism of cell accumulation in these other models of neurodegenerative diseases. Figure 1 summarizes the above-mentioned findings regarding the functional roles of CD14^+^ monocytes, DC, macrophages and microglia in traumatic CNS injury and associated BBB breach such as intracortical electrode implantation.

## Intravascular Cellular Dynamics

While our understanding of the role of different immune subsets involved in implantable bio-material device failure will continue to evolve, one constant parameter is the absolute requirement for circulating cells to successfully navigate the vast vasculature as they traverse and home to sites of insult; either in the periphery or in the CNS. When combining 2P-LSM with resonance scanning, we were able to visualize cellular flow within post-capillary venules in the CNS in real time (Benson et al., 2018). Our initial intravital observations revealed that cellular arrest was most prominent in areas of sudden vasculature volume expansion. As such, we designed biomimetic microfluidic devices that allowed efficient modeling of the CNS vasculature with different characteristic widths. Once coupled with a physiologic hematocrit, purified leukocytes exhibited slow rolling and adhesion that was dependent on Intracellular adhesion molecule-1 (ICAM-1). The modeling of CNS vasculature with microfluidic devices and microscopy allowed us to demonstrate the critical importance of dynamic vessel geometry changes in promoting recruitment of peripheral blood leukocytes. This is likely to critically influence the early response to device implantation, since vessel dilatation is seen as an early response to implantation (Wellman and Kozai, 2018; Eles et al., 2019). Parallel work in suppressing vascular remodeling in lung inflammation suggests angiopoietin 2 (Ang2) as a potential additional target for this process (Le et al., 2015), in addition to TNF which is already known to contribute to gliosis and neuronal death after implantation.

## Additional Considerations

Several recent studies have implicated a potential new role for the deep cervical lymph nodes in regulating CNS immunity (de Vos et al., 2002; Furtado et al., 2008; Hatterer et al., 2008). Until recently, it was believed that the CNS lacked an overt lymphatic system. However, confocal, electron, and intravital 2P-LSM microscopy have now been used to validate the presence and functionality of lymphatic vessels running along the venous sinuses to the deep cervical lymph nodes (Louveau et al., 2015). This knowledge may impact our ongoing investigation into CNS tissue response to injury and repair, which is an issue critical to the field of neuro-electronic interface, device designs and clinical applications. Whether these lymphatic vessels work alone or in concert with other proposed mechanisms of antigen and solute drainage remains to be seen (Bradbury et al., 1981; Walter et al., 2006; Carare et al., 2008; Jessen et al., 2015). Deeper understanding of the newly discovered lymphatic flow within the CNS may inform future decisions as to how neuro-electronic devices should be implanted for best long-term clinical outcome by avoiding immune-mediated tissue repair and inflammatory responses that tend to diminish device functionality over time *in vivo* (McConnell et al., 2009).

## Conclusion

The above insights from 2P-LSM intravital imaging provide several key characteristics to consider for proper design and insertion of implantable bio-materials that could preserve functional integrity without interference from intrinsic host tissue-derived immune response. First, the possibility that resident microglia can participate in direct antigen presentation by extending their processes into intact blood vessels provides a potential mechanism for recruiting and mediating additional peripheral immune cell-CNS parenchyma contact. This argues that while coating of electrodes with non-immunogenic materials and rationale device design may decrease local inflammation, the addition of systemic therapy aimed at reducing inflammatory co-factors in the peripheral circulation may provide added benefit in prolonging device longevity. Additionally, considering that microglia are more concentrated in white matter (Mittelbronn et al., 2001), surgical planning for device insertion through tracks that reduce contact with these immune cells may prove useful in preventing acute and chronic device-associated inflammation. Second, our direct visualization of CNS blood vessel leaks provides a new challenge to consider within the realm of BBB integrity preservation during neuro-electronic device insertion. Understanding regulation of blood vessel leaks and phagocyte activation in damaged CNS microenvironment is critical for future electrode design, as well as our basic understanding of CNS pathology. Our research suggests that treating patients with anti-histamines, i.e., hydroxyzine, may help quench these initial leaks and restrain the initial immune reaction during device implantation. Additionally, recent studies demonstrated that reducing specific clotting factors and bradykinin have beneficial effects in AD. Therefore, a multimodal approach should be considered. For example, administration of icatibant, a bradykinin antagonist approved to treat hereditary angioedema, may be beneficial in the perioperative period. These observations could be extrapolated to basic medication management. For example, ACE inhibitors, with their bradykinin increasing effects, should be avoided in patients with AD or other bio-material implants. Lastly, our new insights from studies using microfluidic devices lend support for treatment with antibodies or small molecules blocking adhesion (ICAM-1) or vessel remodeling (TNF and Ang2) in the perioperative period, as such approaches may have the potential to limit device immunogenicity.

As tissue reactions and immune responses are highly sensitive to tissue trauma within the CNS, care must be taken in conducting experiments where disruption of CNS tissues is involved [such as insertion of glass guide tubing following cortical tissue removal for deep tissue imaging (Barretto et al., 2011; Barretto and Schnitzer, 2012a, b)] in order to allow unbiased interpretation of data. Simple acts of thinning the skull or creation of cranial windows can lead to substantial immune activation and BBB disruption (Dorand et al., 2014). New imaging modalities that allows reduced invasiveness of the CNS tissue, such super-long-wave two-photon microscopy (Kondo et al., 2017), 3-photon microscopy (Liu et al., 2019a, b) or a combination of both techniques (Weisenburger et al., 2019) may offer advantages and opportunities in this regard. As our knowledge of CNS immunology continues to develop, further strategies for limiting immunogenicity of implantable bio-materials could be developed to enhance and sustain their long-term viability. While we continue to advance our knowledge into the world of CNS neuro-immunology, ongoing collaborations with immunologists and neuroscientists will be crucial to optimize future device performance and patient outcome.

## Author Contributions

All authors listed have made a substantial, direct and intellectual contribution to the work, and approved it for publication.

## Conflict of Interest Statement

This work was supported in part by a Sponsored Research Agreement from Biogen Idec.
